# 2-Oxo-2*H*-chromen-4-yl 4-*tert*-butyl­benzoate

**DOI:** 10.1107/S160053681200298X

**Published:** 2012-01-31

**Authors:** Akoun Abou, Bintou Sessouma, Abdoulaye Djandé, Adama Saba, Rita Kakou-Yao

**Affiliations:** a Laboratoire de Cristallographie et Physique Moléculaire, UFR SSMT, Université de Cocody, 22 BP 582, Abidjan 22, Cote d’Ivoire; b Laboratoire de Chimie Bio-organique et Phytochimie, Université de Ouagadougou, 03 BP 7021, Ouagadougou 03, Burkina Faso

## Abstract

In the title mol­ecule, C_20_H_18_O_4_, the three methyl groups of the *tert*-butyl substituent show rotational disorder. Each methyl group is split over three positions, with refined site-occupation factors of 0.711 (4), 0.146 (3) and 0.144 (4). The benzene ring of the benzoate group is oriented at a dihedral angle of 60.70 (7)° with respect to the planar chromene ring [maximum deviation = 0.046 (2) Å]. The crystal structure features centrosymmetric *R*_2_^2^(8) dimers formed *via* C—H⋯O inter­actions, and these dimeric aggregates are connected by C—H⋯π inter­actions.

## Related literature

For the biological activities of coumarin derivatives, see: Ukhov *et al.* (2001 ▶); Abd Elhafez *et al.* (2003 ▶); Basanagouda *et al.* (2009 ▶); Liu *et al.* (2008 ▶); Trapkov *et al.* (1996 ▶); Vukovic *et al.* (2010 ▶); Emmanuel-Giota *et al.* (2001 ▶); Hamdi & Dixneuf (2007 ▶); Wang *et al.* (2001 ▶); Marchenko *et al.* (2006 ▶). For hydrogen-bond motifs, see: Bernstein *et al.* (1995 ▶).
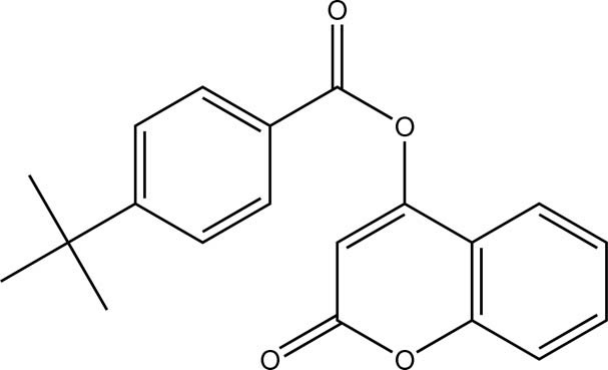


## Experimental

### 

#### Crystal data

C_20_H_18_O_4_
*M* *_r_* = 322.34
Triclinic, 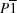

*a* = 6.4319 (2) Å
*b* = 9.3498 (3) Å
*c* = 14.5505 (5) Å
α = 98.481 (1)°
β = 93.655 (1)°
γ = 102.359 (2)°
*V* = 841.27 (5) Å^3^
*Z* = 2
Mo *K*α radiation
μ = 0.09 mm^−1^
*T* = 298 K
0.50 × 0.30 × 0.14 mm

#### Data collection

Nonius KappaCCD diffractometer
11164 measured reflections
4198 independent reflections
2926 reflections with *I* > 2σ(*I*)
*R*_int_ = 0.033

#### Refinement

*R*[*F* ^2^ > 2σ(*F* ^2^)] = 0.057
*wR*(*F* ^2^) = 0.157
*S* = 1.05
4198 reflections
247 parameters
10 restraints
H-atom parameters constrained
Δρ_max_ = 0.18 e Å^−3^
Δρ_min_ = −0.16 e Å^−3^

### 

Data collection: *COLLECT* (Hooft, 1998 ▶); cell refinement: *DENZO* and *SCALEPACK* (Otwinowski & Minor, 1997 ▶); data reduction: *DENZO* and *SCALEPACK*; program(s) used to solve structure: *SIR2004* (Burla *et al.*, 2005 ▶); program(s) used to refine structure: *SHELXL97* (Sheldrick, 2008 ▶); molecular graphics: *PLATON* (Spek, 2009 ▶); software used to prepare material for publication: *SHELXL97*, *publCIF* (Westrip, 2010 ▶) and *WinGX* (Farrugia, 1999 ▶).

## Supplementary Material

Crystal structure: contains datablock(s) I, global. DOI: 10.1107/S160053681200298X/bh2408sup1.cif

Structure factors: contains datablock(s) I. DOI: 10.1107/S160053681200298X/bh2408Isup2.hkl

Supplementary material file. DOI: 10.1107/S160053681200298X/bh2408Isup3.cml

Additional supplementary materials:  crystallographic information; 3D view; checkCIF report

## Figures and Tables

**Table 1:** Hydrogen-bond geometry (Å, °) *Cg*2 and *Cg*3 are the centroids of the chromene benzene and benzoate benzene rings.

*D*—H⋯*A*	*D*—H	H⋯*A*	*D*⋯*A*	*D*—H⋯*A*
C2—H2⋯O2^i^	0.93	2.39	3.323 (2)	177
C18*B*—H18*D*⋯*Cg*3^ii^	0.96	2.83	3.54 (2)	133
C18*C*—H18*I*⋯*Cg*3^ii^	0.96	2.90	3.47 (2)	119
C19*C*—H19*I*⋯*Cg*2^iii^	0.96	2.95	3.75 (2)	141

Symmetry codes: (i) 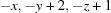; (ii) 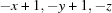; (iii) 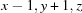.

**Fig. 1.**
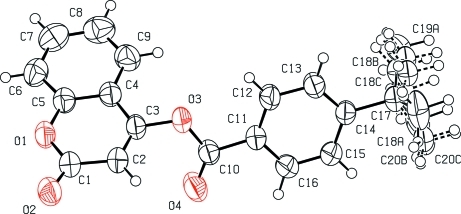
The molecular structure of the title compund, showing displacement ellipsoids at the 50% probability level. H atoms are shown as spheres of arbitrary radius.

**Fig. 2.**
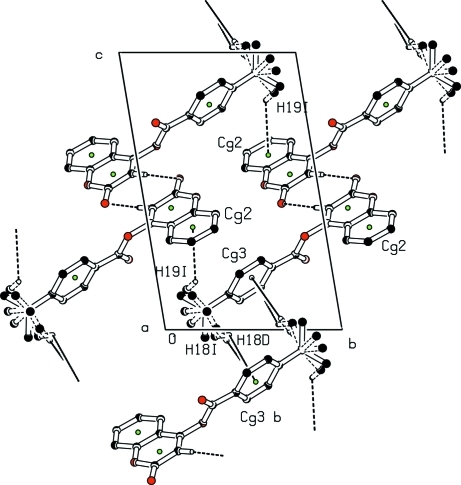
Crystal packing, viewed down the a axis, showing centrosymmetric dimers linked by C—H···π interactions. The green dots are centroids of rings and the dashed lines indicate hydrogen bonds. H atoms not involved in hydrogen bonds have been omitted for clarity.

**Fig. 3.**
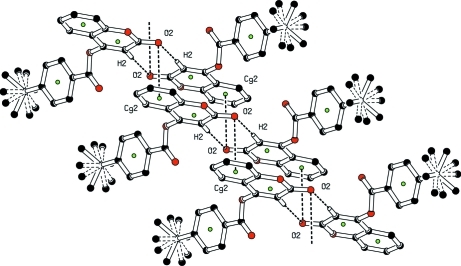
Crystal packing, showing parallel centrosymmetric dimers linked by C═O···π interactions. The green dots are centroids of rings and the dashed lines indicate hydrogen bonds and O···π contacts. H atoms not involved in hydrogen bonds have been omitted for clarity.

## supplementary crystallographic information

### Comment

Coumarin constitutes one of the major classes of naturally occurring compounds, and interest in its chemistry continues unabated because of its usefulness as biologically active agents. It also represents the core structure of several molecules of pharmaceutical importance. Coumarin and its derivatives have been reported to serve as anti-bacterial (Ukhov *et al.*, 2001; Abd Elhafez *et al.*, 2003; Basanagouda *et al.*, 2009; Liu *et al.*, 2008), anti-oxidant (Trapkov *et al.*, 1996; Vukovic *et al.*, 2010), anti-inflammatory (Emmanuel-Giota *et al.*, 2001; Hamdi & Dixneuf, 2007), anti-coagulant (Hamdi & Dixneuf, 2007) and anti-tumour (Wang *et al.*, 2001; Marchenko, *et al.*, 2006) agents. Therefore, the synthesis of new coumarin derivatives is of considerable interest. In order to study the influence of new substituents on the activity of the coumarin derivatives, the title compound has been synthesized and in this paper, we present its molecular and crystal structure.

In the title compound (Fig. 1), the three methyl groups of the *tert*-butyl substituent exhibit rotational disorder, with refined site occupation factors of 0.711 (4), 0.146 (3) and 0.144 (4). The planar chromene ring system resulting from the two fused rings (benzene and 3,6-dihydro-2*H*-pyran) is oriented with respect to the benzoate-benzene ring at a dihedral angle of 60.70 (7)°.

In the crystal structure, intermolecular C—H···O interactions (Table 1) link the molecules into centrosymmetric dimers through *R*_2_^2^(8) ring motifs (Bernstein *et al.*, 1995) and these dimeric aggregates are connected by C—H···π and weak C═O···π interactions (Table 1, Fig. 2 and 3).

### Experimental

To a solution of 4-tertiobutylbenzoyl chloride (4.10^-2^ mole) in dried tetrahydrofuran (150 ml), was added dried triethylamine (0.12 mole) and 4-hydroxycoumarin (4.10^-2^ mole) by small portions over 30 min. The mixture was then refluxed for 3 h and poured in 300 ml of chloroform. The solution was acidified with dilute hydrochloric acid until the pH was 2–3. The organic layer was extracted, washed with water, dried over MgSO_4_ and the solvent removed. The crude product was recrystallized from chloroform. Colourless crystals of the title compound were obtained in good yield 73.8%; melting point: 381–383 K.

### Refinement

In the refinement, positional, site occupation factors and *U*_ij_ parameters of the disordered C atoms were refined freely. However, *EADP* instruction (Sheldrick, 2008) was used to constrain the anisotropic displacement parameters (ADPs) of the disordered C atoms of the two minor components to be the same as their corresponding C atoms in the principal component. Also, *SADI* and *SAME* restrictions were applied to *C*(methyl)···*C*(methyl) separations in each component, in order to get a sensible geometry. H atoms were placed in calculated positions [C—H = 0.93 (aromatic) or 0.96 Å (methyl group)] and refined using a riding model approximation with *U*_iso_(H) constrained to 1.2 (aromatic) or 1.5 (methyl) times *U*_eq_ of the respective parent atom. Four reflections were omitted from the refinement because of large disagreements: (0 0 1), (0 1 0), (0 - 1 1) and (-1 4 6).

### Crystal data

**Table:**

C_20_H_18_O_4_	*Z* = 2
*M**_r_* = 322.34	*F*(000) = 340
Triclinic, *P*1	*D*_x_ = 1.273 Mg m^−^^3^
Hall symbol: -P 1	Melting point = 381–383 K
*a* = 6.4319 (2) Å	Mo *K*α radiation, λ = 0.71073 Å
*b* = 9.3498 (3) Å	Cell parameters from 11164 reflections
*c* = 14.5505 (5) Å	θ = 2.8–29.0°
α = 98.481 (1)°	µ = 0.09 mm^−^^1^
β = 93.655 (1)°	*T* = 298 K
γ = 102.359 (2)°	Parallelepiped, colourless
*V* = 841.27 (5) Å^3^	0.50 × 0.30 × 0.14 mm

### Data collection

**Table:**

Nonius KappaCCD diffractometer	2926 reflections with *I* > 2σ(*I*)
Radiation source: fine-focus sealed tube	*R*_int_ = 0.033
graphite	θ_max_ = 29.0°, θ_min_ = 2.8°
φ and ω scans	*h* = −8→8
11164 measured reflections	*k* = −12→12
4198 independent reflections	*l* = −19→19

### Refinement

**Table:**

Refinement on *F*^2^	Primary atom site location: structure-invariant direct methods
Least-squares matrix: full	Secondary atom site location: difference Fourier map
*R*[*F*^2^ > 2σ(*F*^2^)] = 0.057	Hydrogen site location: inferred from neighbouring sites
*wR*(*F*^2^) = 0.157	H-atom parameters constrained
*S* = 1.05	*w* = 1/[σ^2^(*F*_o_^2^) + (0.0589*P*)^2^ + 0.1855*P*] where *P* = (*F*_o_^2^ + 2*F*_c_^2^)/3
4198 reflections	(Δ/σ)_max_ < 0.001
247 parameters	Δρ_max_ = 0.18 e Å^−^^3^
10 restraints	Δρ_min_ = −0.16 e Å^−^^3^
108 constraints	

### Special details

**Table:**

Refinement. In the title coumpound, the *tert*-butyl group may rotate virtually freely at least at room temperature, and in the spatial average one sees this group as a rotational toroid. Since it is hard to describe this situation to the refinement program, we have reduced the problem to a refinement of only three sites per methyl group (see *Refinement* section). The low *U*_eq_ as compared to neighbors for atom C17 is caused by this disorder.

### Fractional atomic coordinates and isotropic or equivalent isotropic displacement parameters (Å^2^)

**Table:**

	*x*	*y*	*z*	*U*_iso_*/*U*_eq_	Occ. (<1)
O1	0.44961 (18)	1.29961 (11)	0.47812 (8)	0.0616 (3)	
C14	0.3413 (2)	0.39183 (15)	0.12787 (10)	0.0501 (3)	
O3	0.41921 (18)	0.87715 (12)	0.33682 (9)	0.0691 (4)	
O2	0.1580 (2)	1.22217 (14)	0.54429 (9)	0.0740 (4)	
C4	0.6027 (2)	1.13127 (16)	0.37549 (10)	0.0534 (4)	
C12	0.4620 (3)	0.60660 (18)	0.24953 (12)	0.0651 (5)	
H12	0.5638	0.6592	0.2978	0.078*	
O4	0.1052 (2)	0.85839 (15)	0.25363 (10)	0.0838 (4)	
C5	0.6076 (3)	1.27317 (17)	0.42286 (11)	0.0545 (4)	
C11	0.2870 (2)	0.66128 (15)	0.22615 (10)	0.0497 (3)	
C16	0.1363 (3)	0.57977 (18)	0.15545 (12)	0.0623 (4)	
H16	0.0149	0.6138	0.1402	0.075*	
C13	0.4863 (3)	0.47264 (18)	0.20086 (12)	0.0671 (5)	
H13	0.6042	0.4362	0.2180	0.080*	
C2	0.2658 (3)	1.04280 (16)	0.43723 (12)	0.0590 (4)	
H2	0.1486	0.9663	0.4403	0.071*	
C10	0.2528 (3)	0.80567 (16)	0.27141 (11)	0.0537 (4)	
C17	0.3724 (3)	0.24640 (16)	0.07275 (11)	0.0551 (4)	
C15	0.1651 (3)	0.44763 (18)	0.10713 (12)	0.0628 (4)	
H15	0.0626	0.3948	0.0592	0.075*	
C6	0.7699 (3)	1.39340 (19)	0.41635 (13)	0.0679 (5)	
H6	0.7691	1.4877	0.4473	0.081*	
C3	0.4194 (2)	1.01742 (16)	0.38416 (11)	0.0550 (4)	
C9	0.7722 (3)	1.1114 (2)	0.32308 (12)	0.0681 (5)	
H9	0.7745	1.0176	0.2919	0.082*	
C1	0.2803 (3)	1.18853 (17)	0.49010 (12)	0.0575 (4)	
C8	0.9354 (3)	1.2308 (3)	0.31767 (14)	0.0803 (6)	
H8	1.0485	1.2174	0.2831	0.096*	
C7	0.9322 (3)	1.3711 (2)	0.36341 (15)	0.0780 (5)	
H7	1.0420	1.4513	0.3581	0.094*	
C18A	0.2652 (7)	0.2152 (4)	−0.0279 (2)	0.0881 (11)	0.711 (4)
H18A	0.3211	0.2965	−0.0593	0.132*	0.711 (4)
H18B	0.1135	0.2042	−0.0269	0.132*	0.711 (4)
H18C	0.2940	0.1256	−0.0604	0.132*	0.711 (4)
C19A	0.6141 (5)	0.2536 (4)	0.0647 (3)	0.0893 (11)	0.711 (4)
H19A	0.6306	0.1656	0.0255	0.134*	0.711 (4)
H19B	0.6857	0.2605	0.1257	0.134*	0.711 (4)
H19C	0.6752	0.3392	0.0381	0.134*	0.711 (4)
C20A	0.2862 (7)	0.1213 (3)	0.1233 (2)	0.0839 (10)	0.711 (4)
H20A	0.1356	0.1128	0.1267	0.126*	0.711 (4)
H20B	0.3579	0.1406	0.1852	0.126*	0.711 (4)
H20C	0.3098	0.0303	0.0902	0.126*	0.711 (4)
C18B	0.392 (4)	0.266 (2)	−0.0247 (12)	0.0881 (11)	0.144 (4)
H18D	0.5084	0.3477	−0.0275	0.132*	0.144 (4)
H18E	0.2615	0.2846	−0.0508	0.132*	0.144 (4)
H18F	0.4180	0.1769	−0.0597	0.132*	0.144 (4)
C19B	0.554 (3)	0.190 (2)	0.1180 (13)	0.0893 (11)	0.144 (4)
H19D	0.5548	0.0926	0.0857	0.134*	0.144 (4)
H19E	0.5330	0.1849	0.1823	0.134*	0.144 (4)
H19F	0.6884	0.2563	0.1144	0.134*	0.144 (4)
C20B	0.154 (3)	0.1210 (15)	0.0767 (14)	0.0839 (10)	0.144 (4)
H20D	0.0333	0.1525	0.0515	0.126*	0.144 (4)
H20E	0.1378	0.1083	0.1403	0.126*	0.144 (4)
H20F	0.1639	0.0284	0.0406	0.126*	0.144 (4)
C18C	0.512 (3)	0.2889 (16)	0.0029 (10)	0.0881 (11)	0.146 (3)
H18G	0.6459	0.3494	0.0330	0.132*	0.146 (3)
H18H	0.4467	0.3441	−0.0364	0.132*	0.146 (3)
H18I	0.5370	0.2016	−0.0342	0.132*	0.146 (3)
C19C	0.465 (3)	0.1613 (18)	0.1414 (11)	0.0893 (11)	0.146 (3)
H19G	0.4762	0.0664	0.1090	0.134*	0.146 (3)
H19H	0.3729	0.1470	0.1902	0.134*	0.146 (3)
H19I	0.6045	0.2170	0.1681	0.134*	0.146 (3)
C20C	0.151 (3)	0.1494 (14)	0.0290 (12)	0.0839 (10)	0.146 (3)
H20G	0.0924	0.1971	−0.0174	0.126*	0.146 (3)
H20H	0.0553	0.1377	0.0769	0.126*	0.146 (3)
H20I	0.1671	0.0537	0.0004	0.126*	0.146 (3)

### Atomic displacement parameters (Å^2^)

**Table:**

	*U*^11^	*U*^22^	*U*^33^	*U*^12^	*U*^13^	*U*^23^
O1	0.0675 (7)	0.0415 (5)	0.0719 (7)	0.0107 (5)	0.0124 (5)	−0.0031 (5)
C14	0.0553 (8)	0.0396 (7)	0.0530 (8)	0.0100 (6)	0.0031 (6)	0.0018 (6)
O3	0.0669 (7)	0.0466 (6)	0.0872 (8)	0.0212 (5)	−0.0081 (6)	−0.0158 (5)
O2	0.0765 (8)	0.0623 (7)	0.0795 (8)	0.0150 (6)	0.0226 (6)	−0.0061 (6)
C4	0.0596 (9)	0.0490 (8)	0.0510 (8)	0.0159 (6)	0.0010 (6)	0.0033 (6)
C12	0.0680 (10)	0.0546 (9)	0.0662 (10)	0.0222 (7)	−0.0162 (8)	−0.0138 (7)
O4	0.0957 (10)	0.0718 (8)	0.0843 (9)	0.0461 (7)	−0.0186 (7)	−0.0141 (7)
C5	0.0616 (9)	0.0469 (8)	0.0546 (8)	0.0136 (6)	0.0032 (7)	0.0064 (6)
C11	0.0569 (8)	0.0403 (7)	0.0510 (8)	0.0121 (6)	0.0050 (6)	0.0031 (6)
C16	0.0559 (9)	0.0595 (9)	0.0694 (10)	0.0220 (7)	−0.0057 (7)	−0.0043 (8)
C13	0.0699 (10)	0.0575 (9)	0.0706 (11)	0.0294 (8)	−0.0168 (8)	−0.0114 (8)
C2	0.0608 (9)	0.0419 (7)	0.0707 (10)	0.0101 (6)	0.0048 (7)	0.0005 (7)
C10	0.0614 (9)	0.0450 (7)	0.0553 (8)	0.0173 (6)	0.0038 (7)	0.0032 (6)
C17	0.0630 (9)	0.0433 (7)	0.0565 (9)	0.0141 (6)	0.0046 (7)	−0.0024 (6)
C15	0.0551 (9)	0.0557 (9)	0.0695 (10)	0.0136 (7)	−0.0081 (7)	−0.0111 (7)
C6	0.0731 (11)	0.0516 (9)	0.0747 (11)	0.0062 (8)	0.0028 (9)	0.0102 (8)
C3	0.0611 (9)	0.0405 (7)	0.0608 (9)	0.0165 (6)	−0.0026 (7)	−0.0036 (6)
C9	0.0693 (10)	0.0744 (11)	0.0607 (10)	0.0247 (9)	0.0060 (8)	−0.0002 (8)
C1	0.0621 (9)	0.0469 (8)	0.0610 (9)	0.0134 (7)	0.0050 (7)	0.0001 (7)
C8	0.0673 (11)	0.1024 (16)	0.0702 (12)	0.0151 (10)	0.0156 (9)	0.0135 (11)
C7	0.0711 (11)	0.0790 (13)	0.0798 (12)	0.0017 (9)	0.0084 (9)	0.0219 (10)
C18A	0.122 (3)	0.077 (2)	0.0622 (15)	0.044 (2)	−0.0175 (19)	−0.0209 (14)
C19A	0.0668 (16)	0.084 (2)	0.106 (3)	0.0231 (15)	0.0170 (16)	−0.0296 (18)
C20A	0.125 (3)	0.0432 (11)	0.086 (2)	0.0221 (15)	0.0319 (19)	0.0047 (13)
C18B	0.122 (3)	0.077 (2)	0.0622 (15)	0.044 (2)	−0.0175 (19)	−0.0209 (14)
C19B	0.0668 (16)	0.084 (2)	0.106 (3)	0.0231 (15)	0.0170 (16)	−0.0296 (18)
C20B	0.125 (3)	0.0432 (11)	0.086 (2)	0.0221 (15)	0.0319 (19)	0.0047 (13)
C18C	0.122 (3)	0.077 (2)	0.0622 (15)	0.044 (2)	−0.0175 (19)	−0.0209 (14)
C19C	0.0668 (16)	0.084 (2)	0.106 (3)	0.0231 (15)	0.0170 (16)	−0.0296 (18)
C20C	0.125 (3)	0.0432 (11)	0.086 (2)	0.0221 (15)	0.0319 (19)	0.0047 (13)

### Geometric parameters (Å, °)

**Table:**

O1—C1	1.374 (2)	C6—C7	1.369 (3)
O1—C5	1.3760 (19)	C6—H6	0.9300
C14—C15	1.380 (2)	C9—C8	1.375 (3)
C14—C13	1.380 (2)	C9—H9	0.9300
C14—C17	1.5322 (19)	C8—C7	1.385 (3)
O3—C10	1.3691 (19)	C8—H8	0.9300
O3—C3	1.3883 (17)	C7—H7	0.9300
O2—C1	1.2048 (19)	C18A—H18A	0.9600
C4—C5	1.395 (2)	C18A—H18B	0.9600
C4—C9	1.398 (2)	C18A—H18C	0.9600
C4—C3	1.435 (2)	C19A—H19A	0.9600
C12—C11	1.376 (2)	C19A—H19B	0.9600
C12—C13	1.390 (2)	C19A—H19C	0.9600
C12—H12	0.9300	C20A—H20A	0.9600
O4—C10	1.1906 (18)	C20A—H20B	0.9600
C5—C6	1.380 (2)	C20A—H20C	0.9600
C11—C16	1.380 (2)	C18B—H18D	0.9600
C11—C10	1.4792 (19)	C18B—H18E	0.9600
C16—C15	1.383 (2)	C18B—H18F	0.9600
C16—H16	0.9300	C19B—H19D	0.9600
C13—H13	0.9300	C19B—H19E	0.9600
C2—C3	1.331 (2)	C19B—H19F	0.9600
C2—C1	1.444 (2)	C20B—H20D	0.9600
C2—H2	0.9300	C20B—H20E	0.9600
C17—C18C	1.442 (16)	C20B—H20F	0.9600
C17—C18B	1.465 (17)	C18C—H18G	0.9600
C17—C20A	1.498 (3)	C18C—H18H	0.9600
C17—C19C	1.533 (17)	C18C—H18I	0.9600
C17—C19B	1.533 (16)	C19C—H19G	0.9600
C17—C18A	1.538 (3)	C19C—H19H	0.9600
C17—C19A	1.554 (3)	C19C—H19I	0.9600
C17—C20C	1.557 (16)	C20C—H20G	0.9600
C17—C20B	1.634 (17)	C20C—H20H	0.9600
C15—H15	0.9300	C20C—H20I	0.9600
			
C1—O1—C5	122.13 (12)	C14—C17—C20B	106.5 (5)
C15—C14—C13	116.78 (13)	C19C—C17—C20B	82.4 (9)
C15—C14—C17	121.34 (13)	C19B—C17—C20B	105.6 (8)
C13—C14—C17	121.88 (13)	C18A—C17—C20B	76.5 (7)
C10—O3—C3	119.42 (12)	C19A—C17—C20B	138.2 (6)
C5—C4—C9	118.27 (15)	C14—C15—C16	121.97 (14)
C5—C4—C3	116.26 (14)	C14—C15—H15	119.0
C9—C4—C3	125.47 (15)	C16—C15—H15	119.0
C11—C12—C13	119.84 (14)	C7—C6—C5	118.66 (17)
C11—C12—H12	120.1	C7—C6—H6	120.7
C13—C12—H12	120.1	C5—C6—H6	120.7
O1—C5—C6	116.91 (14)	C2—C3—O3	122.37 (15)
O1—C5—C4	121.30 (14)	C2—C3—C4	122.51 (14)
C6—C5—C4	121.79 (16)	O3—C3—C4	115.05 (14)
C12—C11—C16	118.93 (13)	C8—C9—C4	119.91 (18)
C12—C11—C10	123.52 (14)	C8—C9—H9	120.0
C16—C11—C10	117.54 (13)	C4—C9—H9	120.0
C11—C16—C15	120.26 (14)	O2—C1—O1	116.63 (14)
C11—C16—H16	119.9	O2—C1—C2	126.09 (16)
C15—C16—H16	119.9	O1—C1—C2	117.28 (14)
C14—C13—C12	122.17 (14)	C9—C8—C7	120.33 (18)
C14—C13—H13	118.9	C9—C8—H8	119.8
C12—C13—H13	118.9	C7—C8—H8	119.8
C3—C2—C1	120.32 (15)	C6—C7—C8	121.00 (18)
C3—C2—H2	119.8	C6—C7—H7	119.5
C1—C2—H2	119.8	C8—C7—H7	119.5
O4—C10—O3	122.60 (14)	C17—C18A—H18A	109.5
O4—C10—C11	126.25 (15)	C17—C18A—H18B	109.5
O3—C10—C11	111.12 (12)	C17—C18A—H18C	109.5
C18C—C17—C20A	143.4 (6)	C17—C19A—H19A	109.5
C18B—C17—C20A	136.4 (7)	C17—C19A—H19B	109.5
C18C—C17—C14	105.8 (5)	C17—C19A—H19C	109.5
C18B—C17—C14	108.4 (6)	C17—C20A—H20A	109.5
C20A—C17—C14	109.06 (15)	C17—C20A—H20B	109.5
C18C—C17—C19C	113.7 (8)	C17—C20A—H20C	109.5
C18B—C17—C19C	136.7 (8)	C17—C18B—H18D	109.5
C14—C17—C19C	108.2 (5)	C17—C18B—H18E	109.5
C18C—C17—C19B	87.6 (9)	H18D—C18B—H18E	109.5
C18B—C17—C19B	114.8 (8)	C17—C18B—H18F	109.5
C20A—C17—C19B	69.0 (8)	H18D—C18B—H18F	109.5
C14—C17—C19B	112.8 (5)	H18E—C18B—H18F	109.5
C18C—C17—C18A	64.7 (7)	C17—C19B—H19D	109.5
C20A—C17—C18A	110.4 (2)	C17—C19B—H19E	109.5
C14—C17—C18A	112.45 (16)	H19D—C19B—H19E	109.5
C19C—C17—C18A	138.0 (5)	C17—C19B—H19F	109.5
C19B—C17—C18A	131.7 (6)	H19D—C19B—H19F	109.5
C18C—C17—C19A	47.3 (7)	H19E—C19B—H19F	109.5
C18B—C17—C19A	78.1 (9)	C17—C20B—H20D	109.5
C20A—C17—C19A	108.4 (2)	C17—C20B—H20E	109.5
C14—C17—C19A	110.35 (15)	H20D—C20B—H20E	109.5
C19C—C17—C19A	67.9 (7)	C17—C20B—H20F	109.5
C18A—C17—C19A	106.2 (2)	H20D—C20B—H20F	109.5
C18C—C17—C20C	112.4 (7)	H20E—C20B—H20F	109.5
C18B—C17—C20C	81.3 (9)	C17—C18C—H18G	109.5
C20A—C17—C20C	65.9 (6)	C17—C18C—H18H	109.5
C14—C17—C20C	109.1 (5)	C17—C18C—H18I	109.5
C19C—C17—C20C	107.5 (7)	C17—C19C—H19G	109.5
C19B—C17—C20C	125.8 (8)	C17—C19C—H19H	109.5
C18A—C17—C20C	48.9 (7)	C17—C19C—H19I	109.5
C19A—C17—C20C	139.5 (5)	C17—C20C—H20G	109.5
C18C—C17—C20B	136.6 (8)	C17—C20C—H20H	109.5
C18B—C17—C20B	108.4 (8)	C17—C20C—H20I	109.5
			
C1—O1—C5—C6	−179.82 (15)	C13—C14—C17—C18A	−152.6 (2)
C1—O1—C5—C4	0.4 (2)	C15—C14—C17—C19A	146.2 (2)
C9—C4—C5—O1	−177.87 (14)	C13—C14—C17—C19A	−34.3 (3)
C3—C4—C5—O1	2.9 (2)	C15—C14—C17—C20C	−24.5 (7)
C9—C4—C5—C6	2.4 (2)	C13—C14—C17—C20C	155.0 (7)
C3—C4—C5—C6	−176.80 (15)	C15—C14—C17—C20B	−54.0 (9)
C13—C12—C11—C16	1.2 (3)	C13—C14—C17—C20B	125.5 (9)
C13—C12—C11—C10	−177.33 (16)	C13—C14—C15—C16	1.4 (3)
C12—C11—C16—C15	−2.0 (3)	C17—C14—C15—C16	−179.10 (16)
C10—C11—C16—C15	176.59 (16)	C11—C16—C15—C14	0.7 (3)
C15—C14—C13—C12	−2.2 (3)	O1—C5—C6—C7	178.80 (16)
C17—C14—C13—C12	178.25 (17)	C4—C5—C6—C7	−1.4 (3)
C11—C12—C13—C14	1.0 (3)	C1—C2—C3—O3	175.81 (15)
C3—O3—C10—O4	−0.7 (3)	C1—C2—C3—C4	−0.9 (3)
C3—O3—C10—C11	177.51 (14)	C10—O3—C3—C2	60.6 (2)
C12—C11—C10—O4	179.64 (18)	C10—O3—C3—C4	−122.45 (16)
C16—C11—C10—O4	1.1 (3)	C5—C4—C3—C2	−2.7 (2)
C12—C11—C10—O3	1.5 (2)	C9—C4—C3—C2	178.21 (16)
C16—C11—C10—O3	−177.09 (14)	C5—C4—C3—O3	−179.61 (13)
C15—C14—C17—C18C	96.6 (8)	C9—C4—C3—O3	1.3 (2)
C13—C14—C17—C18C	−83.9 (8)	C5—C4—C9—C8	−1.4 (3)
C15—C14—C17—C18B	62.4 (10)	C3—C4—C9—C8	177.69 (17)
C13—C14—C17—C18B	−118.1 (10)	C5—O1—C1—O2	175.72 (14)
C15—C14—C17—C20A	−94.8 (2)	C5—O1—C1—C2	−4.0 (2)
C13—C14—C17—C20A	84.7 (3)	C3—C2—C1—O2	−175.46 (17)
C15—C14—C17—C19C	−141.2 (7)	C3—C2—C1—O1	4.2 (2)
C13—C14—C17—C19C	38.2 (8)	C4—C9—C8—C7	−0.4 (3)
C15—C14—C17—C19B	−169.4 (10)	C5—C6—C7—C8	−0.5 (3)
C13—C14—C17—C19B	10.1 (10)	C9—C8—C7—C6	1.4 (3)
C15—C14—C17—C18A	27.9 (3)		

### Hydrogen-bond geometry (Å, °)

**Table:**

*D*—H···*A*	*D*—H	H···*A*	*D*···*A*	*D*—H···*A*
C2—H2···O2^i^	0.93	2.39	3.323 (2)	177
C18B—H18D···Cg3^ii^	0.96	2.83	3.54 (2)	133
C18C—H18I···Cg3^ii^	0.96	2.90	3.47 (2)	119
C19C—H19I···Cg2^iii^	0.96	2.95	3.75 (2)	141
C1—O2···Cg2^iv^	1.205 (2)	3.528 (2)	3.802 (2)	94.68 (11)

Symmetry codes: (i) −*x*, −*y*+2, −*z*+1; (ii) −*x*+1, −*y*+1, −*z*; (iii) *x*−1, *y*+1, *z*; (iv) *x*−1, *y*, *z*.
